# Arf6-Dependent Intracellular Trafficking of *Pasteurella multocida* Toxin and pH-Dependent Translocation from Late Endosomes

**DOI:** 10.3390/toxins3030218

**Published:** 2011-03-16

**Authors:** Tana L. Repella, Mengfei Ho, Tracy P. M. Chong, Yuka Bannai, Brenda A. Wilson

**Affiliations:** 1 Department of Microbiology, University of Illinois at Urbana-Champaign, 601 South Goodwin Avenue, Urbana, IL 61801, USA; Email: repella@illinois.edu (T.L.R.); mengho@life.illinois.edu (M.H.); chong@illinois.edu (T.P.M.C.); bannai@illinois.edu (Y.B.); 2 Host-Microbe Systems Theme of the Institute for Genomic Biology, University of Illinois at Urbana-Champaign, 601 South Goodwin Avenue, Urbana, IL 61801, USA

**Keywords:** intoxication, translocation, endocytosis

## Abstract

The potent mitogenic toxin from *Pasteurella multocida* (PMT) is the major virulence factor associated with a number of epizootic and zoonotic diseases caused by infection with this respiratory pathogen. PMT is a glutamine-specific protein deamidase that acts on its intracellular G-protein targets to increase intracellular calcium, cytoskeletal, and mitogenic signaling. PMT enters cells through receptor-mediated endocytosis and then translocates into the cytosol through a pH-dependent process that is inhibited by NH_4_Cl or bafilomycin A1. However, the detailed mechanisms that govern cellular entry, trafficking, and translocation of PMT remain unclear. Co-localization studies described herein revealed that while PMT shares an initial entry pathway with transferrin (Tfn) and cholera toxin (CT), the trafficking pathways of Tfn, CT, and PMT subsequently diverge, as Tfn is trafficked to recycling endosomes, CT is trafficked retrograde to the ER, and PMT is trafficked to late endosomes. Our studies implicate the small regulatory GTPase Arf6 in the endocytic trafficking of PMT. Translocation of PMT from the endocytic vesicle occurs through a pH-dependent process that is also dependent on both microtubule and actin dynamics, as evidenced by inhibition of PMT activity in our SRE-based reporter assay, with nocodazole and cytochalasin D, respectively, suggesting that membrane translocation and cytotoxicity of PMT is dependent on its transfer to late endosomal compartments. In contrast, disruption of Golgi-ER trafficking with brefeldin A increased PMT activity, suggesting that inhibiting PMT trafficking to non-productive compartments that do not lead to translocation, while promoting formation of an acidic tubulovesicle system more conducive to translocation, enhances PMT translocation and activity.

## 1. Introduction

Toxinogenic strains of *Pasteurella multocida* are associated with a number of epizootic and zoonotic diseases, including atrophic rhinitis in swine, and respiratory disease and pasteurellosis in rabbits, cattle, and other domestic and wild animals (reviewed in [1,2]). In humans, *P. multocida* is associated with dermonecrosis and bacteremia from animal bite wounds or respiratory infections from chronic zoonotic exposure to infected livestock or pets (e.g., rabbits, cats, dogs) [3]. The 1285-amino acid, multi-functional protein toxin produced by *P. multocida* serotype A and D strains (PMT) experimentally induces all of the major symptoms of these diseases [4], and has been proposed as a contributor to post-exposure bone loss [1,5], weight reduction and fat loss [6,7,8], immune modulation [9], and potential cancer development [8,10].

Intoxication of mammalian cells by PMT activates a number of different intracellular calcium, cytoskeletal, and mitogenic (MAPK and JAK/STAT) signaling pathways (reviewed in [11]) through the activity of its *C*-terminal C3 domain [12,13], which acts as a glutamine-specific deamidase of its targeted heterotrimeric G-protein α subunits [14]. Since PMT activates several mitogenic signaling pathways that lead to downstream activation of serum response element (SRE) transcription [15,16], it is possible to use an SRE-based luciferase reporter system to measure intracellular activity of PMT [13].

Although the intracellular action of PMT is becoming more defined [17], little is known about the trafficking pathways the toxin exploits to gain access to its cytosolic targets. The lag time between toxin treatment and toxin activity supports the assertion that PMT is an intracellularly-acting toxin [18]. In addition, it has been demonstrated that the addition of PMT-specific antibodies or the addition of weak bases, such as methylamine, only inhibit PMT activity if added during the early stages of intoxication [18]. Furthermore, exposure of cells to PMT at 4 °C results in no detectable PMT activity [18], suggesting that endocytic trafficking involving a pH-dependent step is necessary for PMT intoxication. This was confirmed by use of bafilomycin A1 (BFA), an inhibitor of the vacuolar ATPase proton pump [19], as measured by its inhibition of PMT-mediated phosphorylation of focal adhesion kinase (FAK) [20]. Furthermore, when PMT was bound to the cell surface and endocytosis was inhibited, there was an increase in PMT activity upon exposing the cells to an acidic pH, suggesting that the drop in pH caused PMT to translocate directly across the plasma membrane [20]. In this same study, PMT was found to insert into lipids in a pH-dependent manner and at a pH similar to that at which the protein is reported to unfold [21]. Together, these data support a model in which PMT is endocytosed and trafficked through a low pH compartment where PMT unfolds, then inserts into and translocates across the vesicle membrane to presumably deliver the catalytic domain into the cytosol. 

The small GTPase Arf6 is involved in trafficking of ligands from the plasma membrane and localizes both to the plasma membrane and to internal punctate structures. Intra-endosomal acidification has been shown to result in recruitment of Arf6, along with its cognate GDP/GTP exchange factor (GEF) and an ADP-ribosylation factor nucleotide site opener (ARNO), to the endosomal membrane [22]. Once at the endosomal membrane, Arf6 binds to the c-subunit of the vacuolar ATPase, while ARNO binds to the a2-isoform [23]. Internal structures that label for Arf6 are tubulovesicular in shape, and Arf6 can be visualized on tubule extensions, suggesting that Arf6 also plays a role in recycling back to the plasma membrane [24]. It has been demonstrated that Arf6 positive vesicles can recruit markers of the early endosome, such as early endosome antigen 1 (EEA1), to their surface [25]. Alternatively, the fusion of primary endocytic vesicles with early endosomes is also regulated by Rab5, another small regulatory GTPase involved in intracellular trafficking of recycling endosomes [26], and EEA1 [27]. This fusion is mediated by the activity of PI-3 kinase, as specific inhibitors of PI-3 kinase such as LY294002 prevent this fusion from occurring [28]. 

Transferrin (Tfn) and its cognate receptor are ubiquitous iron-uptake proteins that are well-characterized markers for Rab5-containing clathrin-coated vesicles and recycling endosomes [29]. The trafficking of Tfn through Arf6 compartments seems to differ according to cell type. In HeLa cells Tfn localizes to a separate compartment from Arf6 [30], while in CHO cells and HEK-293T cells transferrin localizes to the same compartment as Arf6 [31,32]. While overexpression of wildtype Arf6 does not affect the function of Arf6 or its distribution and localization in cells, specific mutations that interfere with GDP/GTP binding and/or hydrolysis do [24]. The constitutively active, GTP-hydrolysis-defective mutants of Arf6 (Q67L) localizes to the plasma membrane and causes a reduction in the formation of endosomes, whereas the dominant negative, GTP-binding-defective Arf6 mutant (T27N) localizes almost exclusively in endosomes [24]. Overexpression of Arf6 Q67L mutant results in an increase in cell surface binding of Tfn, but also in a decrease in the rate of internalization of Tfn [33], suggesting that while the mutant results in an increase in binding of the ligand it is unable to subsequently internalize it. Alternatively, overexpression of the Arf6 T27N mutant results in a decrease in the amount of cell surface-bound Tfn and prevents reappearance of cell surface Tfn receptors [33]. 

Cholera toxin (CT), a multi-subunit protein toxin secreted by *Vibrio cholerae*, has been shown to recruit Arf6 to endosomal membranes during its cellular intoxication process [34]. Arf6 also serves as an allosteric activator of the CT-catalyzed ADP-ribosylation of Gα_s_ [35,36], with crystal structures of an Arf6-CT complex lending insight into how this occurs through changes in the active site loop structure that facilitates NAD binding [37]. Furthermore, CT has been shown to increase rates of intra-endosomal acidification [34] and while intra-endosomal acidification is not thought to be important for translocation of CT, the trafficking of CT through a low pH compartment has been demonstrated to be important for proteolytic activation of the catalytic subunit [38]. These observations suggest the possibility that PMT may use the same initial trafficking pathway as Tfn and/or CT, and may be trafficked to an acidified Arf6-positive endosome, from where it may translocate. 

Although we hypothesize that PMT shares a common initial entry pathway with CT, CT has a KDEL signal and undergoes further retrograde trafficking through the Golgi to the endoplasmic reticulum (ER), where the catalytic subunit of CT escapes to the cytosol [39]. It has been demonstrated that treatment with brefeldin A (BFA), a fungal metabolite that interrupts trafficking between the Golgi apparatus and the ER [40], disrupts CT activity implicating the importance of Golgi-ER trafficking for CT [41]. Since we propose that PMT translocates from the acidified endosome, treatment with BFA should have no effect on PMT activity and would provide a useful tool for discerning where the entry pathways of PMT and CT diverge. On the other hand, although PMT translocation is pH dependent, it is not clear whether translocation occurs from early endosomes or late endosomes. Since it has been shown that vectoral transport from early to late endosomes is dependent on microtubule and actin dynamics [27,42], disruption of the microtubule network with nocodazole or actin polymerization with cytochalasin D should inhibit PMT translocation and cytotoxicity.

Our studies reported herein aim to elucidate the trafficking events involved in PMT intoxication. In particular, we describe the role of the small GTPase Arf6 in the uptake and trafficking of PMT. Furthermore, we show that PMT shares the same initial pathway for entry into the cell as Tfn and CT, but then the PMT trafficking pathway diverges from the others to a late-endosomal compartment, from which PMT then translocates in a pH-dependent manner.

## 2. Materials and Methods

### 2.1. Plasmid Constructs

The pcDNA3.1 vector encoding the wild-type Arf6 protein was obtained from the Missouri S&T cDNA Resource Center. pcDNA3.1-Arf6 T27N, pcDNA3.1-Arf6 T44N, pcDNA3.1-Arf6 Q67L, pcDNA3.1-Arf6 T157A, pcDNA3.1-Arf6 G2A/Q67L, and pcDNA3.1-Arf6 G2A/T157A mutants were created by a two step PCR with the first step introducing the mutation and a second step amplifying the entire cDNA using the primers listed in Table 1. The cDNA was then digested with XhoI and EcoRI and inserted into the pcDNA3.1 vector. Constructs were verified by sequencing.

**Table 1:** Primers used in construction of Arf6 mutants.

Primer Name	Sequence (5' to 3')
Arf6 T27N	CGCGGCCGGCAAGAACACAATCCTGTACAAGTTG
Arf6 T27Nr	CAACTTGTACAGGATTGTGTTCTTGCCGGCCGCG
Arf6-T44N	GACCACCATTCCCAATGTGGGTTTCAACGT
Arf6-T44Nr	ACGTTGAAACCCACATTGGGAATGGTGGTC
Arf6 Q67L	ATGTGGGCGGCCTGGACAAGATCCG
Arf6 Q67Lr	CGGATCTTGTCCAGGCCGCCCACAT
Arf6 Q67L 2	TATGGGATGTGGGCGGCCTGGACAAGATCCGGCCGCTCTG
Arf6 Q67Lr 2	CAGAGCGGCCGGATCTTGTCCAGGCCGCCCACATCCCATA
Arf6 T157A	TCCTGTGCCGCCTCAGGGGACG
Arf6 T157Ar	CGTCCCCTGAGGCGGCACAGGA
Arf6 G2A	GTGGAATTCACCATGGCGAAGGTGCTATCCAAAATCTTC
Arf6 G2Ar	GAAGATTTTGGATAGCACCTTCGCCATGGTGAATTCCA
pcDNAF	CGCAAATGGGCGGTAGGCGTG
BGHr2	CAACAGATGGCTGGCAAC

### 2.2. Expression, Purification and Quantification of PMT and PMTb-GFP

Recombinant PMT (rPMT) was expressed and purified as previously described [13]. rPMT was expressed in *Escherichia coli* TOP10 cells (Invitrogen) in the pTHC-ToxA vector under the induction of IPTG. The cell extract was purified by Ni^2+^-NTA-agarose chromatography (Qiagen). Fractions containing rPMT were further purified by FPLC using HiTrapQ anion exchange chromatography (Amersham) and desalted with a PD-10 column (Amersham). Removal of the His_6_-tag was accomplished according to manufacturer’s protocol using a Thrombin Cleavage Capture Kit (Novagen) and rPMT was further purified by FPLC using a HiTrapQ anion exchange column and a Superdex 200 sizing column. Fractions containing rPMT were concentrated using Centricon filter units (Millipore) and desalted using a PD-10 column with phosphate-buffered saline (PBS) containing 10% glycerol. The concentration of rPMT was determined by NIH Image J digital image analysis of Pierce GelCode Blue-stained SDS-PAGE gels using BSA as the standard. Toxin samples were stored at −80 °C until use.

An *N*-terminal fragment of PMT (residues 1–568) with GFP at the *C*-terminus (denoted as PMTb-GFP) was expressed in BL21 cells (Novagen) in the pET21b vector (Novagen). The cell extract was purified by Ni^2+^-NTA-agarose chromatography (Qiagen). Fractions containing rPMT were further purified by FPLC using HiTrapQ anion exchange chromatography (Amersham) and desalted with a PD-10 column (Amersham). Concentration was determined as described above.

### 2.3. Cell Culture

HEK 293-T cells (ATCC # CRL-11268) and Swiss 3T3 cells (ATCC # CCL-92) were cultured and maintained at 37 °C and 5% CO_2_ in DMEM (Gibco) with 10% fetal bovine serum (FBS), 100 U/mL penicillin G, and 100 µg/mL streptomycin.

### 2.4. SRE Assay

HEK 293-T cells at 80% confluency were replated at a 1:7 ratio in 24 well plates. The next day the medium was changed to DMEM with 2% FBS, penicillin and streptomycin, and cells were transfected using the CaCl_2_ method [43]. The plasmid DNA (0.25 µg/mL of pSRE-*luc* (Stratagene), 0.025 µg/mL *p*-Renilla-TK (pGL 7.4 hRluc/TK, Promega), 0.025 µg/mL pcDNA3-Gα_q_ and 0.1 µg/mL of a vector encoding the protein of interest) in a solution of 250 mM CaCl_2_ was added dropwise to a solution of 2× HEPES-buffered saline while vortexing. The solution was incubated at room temperature for 20 min and then added dropwise to each well. Cells were incubated for 7 h, after which the medium was changed without or with rPMT (at a final concentration of 100 ng/mL). After 16 h of rPMT treatment the medium was removed, and cells were lysed by adding 150 µL of 1× Passive Lysis Buffer (Promega) and incubating for 15 min on a rotary shaker. Cell lysates were analyzed using the Dual Luciferase Assay System (Promega), according to manufacturer’s protocol. Luminescence was measured using a Synergy-HT multi-detection microplate reader (BioTek) and results were reported as relative light units (sensitivity = 100, integration time = 1 s).

### 2.5. Data Analysis

SRE activity was determined by dividing the firefly luciferase activity by the *Renilla* luciferase activity. Within each experiment SRE activity was averaged and the average of the rPMT treated was divided by the average of the untreated SRE activity to determine the fold activation. In the Arf6 overexpression experiments the fold activation for the test vector was then divided by fold activation of the empty vector to obtain the fold activation, normalized to control. In the Arf6 experiments, data is expressed as the mean ± S.D. of results from eight independent experiments repeated in triplicate. In the chemical inhibitor experiments, data is expressed as the mean ± S.D. of results from three independent experiments repeated in triplicate. A student’s t-test was then used to compare the fold activation values of each test vector to the empty vector control in the Arf6 experiments and to compare the fold activation values of the treated and untreated in the experiments using chemical inhibitors.

### 2.6. Treatment of Cells with Toxins and Inhibitors

HEK 293-T cells at 80% confluency, maintained as described above, were plated at a 1:7 ratio in 24-well plates. The next day the medium was changed to DMEM with 2% FBS, penicillin and streptomycin, and cells were transfected using the CaCl_2_ method, as described above. Each well was transfected with 250 ng/mL of pSRE-*luc* (Stratagene), 25  ng /mL *p*-Renilla-TK (pGL 7.4 hRluc/TK, Promega), and 25 ng/mL pcDNA3-Gα_q_. Cells were incubated for 7 h at 37 °C after which medium containing the indicated inhibitor was added to the wells. Stock solutions of 700 nM bafilomycin A1 (BAF) (Alexis Biochemicals), 200 µM cytochalasin D (Sigma), 100 µM nocodazole (Sigma), and 700 µM LY294002 (Alexis Biochemicals) were created by dissolving the inhibitor in DMSO. A stock solution of 140 mM NH_4_Cl (J. T. Baker) was created by dissolving NH_4_Cl in water. The stock solution of 70 µM brefeldin A (BFA) (Alexis Biochemicals) was created by dissolving the inhibitor in methanol. After 15 min incubation with the inhibitor, rPMT was added to the wells at a final concentration of 100 ng/mL. After 16 h of rPMT treatment the media was removed and the cells were lysed by adding 150 µL of 1× Passive Lysis Buffer (Promega) and incubating for 15 min on a rotary shaker. Cell lysates were analyzed using the Dual Luciferase Assay System (Promega), according to the manufacturer’s protocol. Luminescence was measured using a Synergy-HT multi-detection microplate reader (BioTek). SRE activity was determined as described above, and the data was expressed as the mean ± S.D. of results from three independent experiments repeated in triplicate.

### 2.7. Western Blot Analysis

HEK 293-T cell lysates were subjected to western blot analysis. Lysates were separated on a 10% SDS-PAGE acrylamide gel and subsequently transferred to a nitrocellulose membrane. Membranes were blocked for 30 min at room temperature in 5% powdered milk in 10 mM Tris-blocking buffer (10 mM Tris-HCl, pH 8.0, 2 mM EDTA, 50 mM NaCl, 0.1% NaI). Membranes were then incubated overnight at 4 °C with the indicated primary antibody in 5% milk in 10 mM Tris-blocking buffer. Membranes were then washed 5 times for 3 min in 1% Tween in 100 mM Tris-washing buffer (100 mM Tris-HCl, pH 8.0, 200 mM NaCl) before incubation at room temperature with the appropriate secondary antibody. The following primary antibodies were used: mouse anti-phospho-Akt (Ser473) 587F11 (4051, Cell Signaling) and mouse anti-Akt (9272, Cell Signaling). HRP-conjugated secondary antibodies used included: goat anti-mouse IgG (115-036-003, Jackson ImmunoResearch Laboratories, Inc.) and goat anti-rabbit (sc-2004, Santa Cruz Biotechnology). After washing 5 times for 3 min each with Tris-washing buffer, the membranes were then developed using Pierce^®^ ECL Western Blotting Substrate (Thermo Scientific) and HyBlot CL autoradiography film (Denville Scientific Inc.). Images were then scanned and prepared using Adobe Photoshop. Blots shown are representative of at least three independent experiments.

### 2.8. Colocalization Studies

Swiss 3T3 cells at 80% confluency were plated in a 1:12 ratio on 18mm circular coverslips (Fisher) in 12 well plates containing DMEM with 1% BGS. The next day the medium was changed 3–4 h before toxin treatment. If chemical inhibitors were used, medium containing the indicated inhibitor, final concentration of 30 mM NH_4_Cl or 1 µM BFA, was added to the wells 15 min before toxin treatment. After 15 min incubation with the inhibitor, PMTb-GFP was added to the wells at a final concentration of 100 µg/mL. CT subunit B Alexa Fluor^®^-594 conjugate (CTxB-594) (Molecular Probes) was added to the wells at a final concentration of 1 µg/mL. After 1 or 3 h of toxin treatment the medium was removed and the coverslips were fixed with 3.7% formaldehyde in DMEM 10% BGS for 20 min. Coverslips were washed 3 times with 1× PBS and mounted onto a No. 1 ½, 22 × 50 mm coverslip (Corning) using ProLong Gold mounting media (Invitrogen). Confocal microscopy was carried out with a Zeiss LSM 710 NLO (Zeiss). Images were generated as described above.

### 2.9. Labeling of the Endosomes with Transferrin-Texas Red

Swiss 3T3 cells were plated at low density (10^3^ cells per well) on coverslips in a 12-well plate and grown overnight in 10% BGS-DMEM. Before toxin treatment, the cells were washed 2 times with 1× PBS containing gentamicin and PMTb-GFP was added to the wells at a final concentration of 100–260 μg/mL of PMTb-GFP. After 15-h incubation, 20 μg/mL of Transferrin-Texas Red (Tfn-TR) was added to each well and incubated for an additional 3.5 h. Toxin treatment was terminated by washing the wells with 1× PBS and fixing the cells with 3.7% formaldehyde in PBS. Coverslips were washed 3 times with 1× PBS and mounted using mounting medium consisting of 1× PBS, 180 mg/mL polyvinyl alcohol (PVA), 27% glycerol and 2 g/L DABCO anti-fade reagent (1,4-diazabicyclo-[2.2.2]octane). Confocal microscopy was performed using a Carl Zeiss Laser Scanning Microscope (LSM 510) with a 40× oil-immersion objective. Images were generated as described above.

## 3. Results and Discussion

### 3.1. Colocalization of PMTb with Tfn

**Figure 1:**
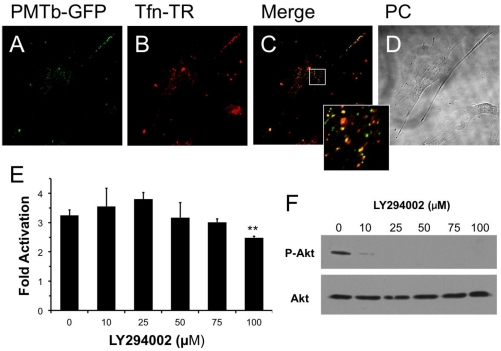
PMTb-GFP initially colocalizes with Tfn-Texas Red, but then diverges and is trafficked to endosomes through a PI3-kinase-independent process. Shown are confocal microscope images of Swiss 3T3 cells after treatment with PMTb-GFP, with the endosomes marked by Transferrin-Texas Red (Tfn-TR). Cells were treated with 260 µg/mL PMTb-GFP and with 20 µg/mL Tfn-TR for 3.5 h to visualize the endosomes. Cells were visualized by confocal microscopy using a 40× objective. (**a**) PMTb-GFP; (**b**) Tfn-TR; (**c**) merged image of (a) and (b); (**d**) corresponding phase-contrast image. Inset: Enlargement of the indicated section of the image, showing co-localization of PMTb-GFP and Tfn-TR; (**e**) HEK 293-T cells were transiently transfected with dual SRE-luciferase reporter plasmids and pcDNA3-Gα_q_ as described in Methods. Seven h post-transfection cells were treated with LY294002 at the indicated concentrations and incubated for 15 min before treatment with 100 ng/mL PMT. After 15 h incubation, cells were harvested and assayed for SRE reporter gene activity, as described in Methods. SRE fold activation was determined by dividing SRE reporter gene activity in PMT-treated cells by SRE reporter gene activity in untreated control cells. Data is expressed as an average of three experiments ± S.D., with each experiment performed in triplicate, where ** *p* < 0.005; (**f**) HEK 293-T cell extracts treated with LY294002 at the indicated concentrations were subjected to western blot analysis to detect P-Akt and total Akt.

A number of bacterial toxins are internalized via receptor-mediated endocytosis, yet the precise pathway taken varies among the different toxins. To determine which pathway is utilized by PMT, we first sought whether PMT is internalized via the same pathway as Tfn, which traffics primarily through Arf6-positive early endosomes [33] and Rab5-associated recycling endosomes [44]. We examined the localization of a GFP-tagged *N*-terminal fragment (residues 1–568) of PMT (PMTb-GFP), which harbors the domains responsible for cellular binding and internalization [45]. We treated Swiss 3T3 fibroblastic cells with PMTb-GFP, and Tfn-TR to visualize colocalization. Both PMTb-GFP and Tfn-TR were distributed in vesicles throughout the cytosol (Figure 1A,B) and appeared to be colocalized within endosomes (Figure 1C).

**Figure 2:**
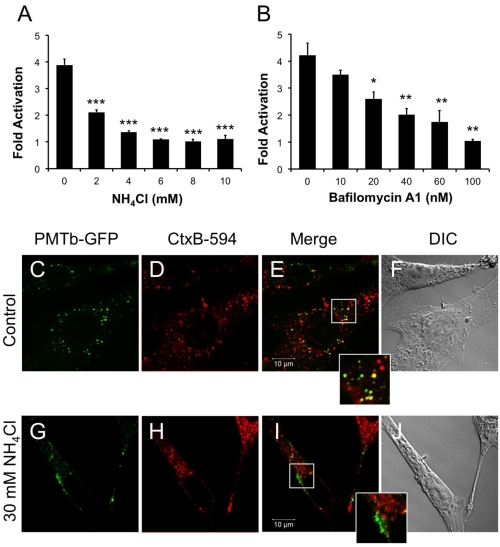
PMTb-GFP initially colocalizes with CTxB-594, but then diverges and is trafficked to and translocated from acidic endosomes. HEK 293-T cells were transiently transfected with dual SRE-luciferase reporter plasmids and pcDNA3-Gα_q_ as described in Methods. Seven h post-transfection cells were treated with (**a**) NH_4_Cl or (**b**) bafilomycin A1 (BAF) at the indicated concentrations for 15 min before treatment with 100 ng/mL rPMT. After 15 h incubation, cells were harvested and assayed for SRE reporter gene activity, as described in the Methods. SRE fold activation was determined by dividing SRE reporter gene activity in PMT-treated cells by SRE reporter gene activity in untreated control cells. Data is expressed as an average of three experiments ± S.D. with each experiment performed in triplicate, where * *p* < 0.05, ** *p* < 0.005, and *** *p* < 0.000005. (**c**–**j**) Swiss 3T3 cells were co-treated with PMTb-GFP and CT subunit B Alexa Fluor^®^-594 conjugate (CtxB-594) for 3 h, as described in Methods, without (**c**–**f**) or with (**g**–**j**) pretreatment for 30 min with 30 mM NH_4_Cl. Cells were visualized by confocal microscopy. Panels (**c**) and (**g**), PMTb-GFP. Panels (**d**) and (**h**), CtxB-594. Panels (**e**) and (**i**), merged images of green and red channels. Panels (**f**) and (**j**), corresponding DIC images. Insets: Enlargement of the indicated section of the image, showing co-localization of PMTb-GFP and CtxB-594.

### 3.2. PI3-Kinase-Mediated Fusion of Arf6-Positive and EEA1/Rab5-Positive Endosomes Is Not Necessary for PMT Intoxication

It has been previously demonstrated that Arf6 is implicated in internalization of Tfn [33]. It is also known that Arf6-positive early endosomes can fuse with Rab5/EEA1-positive endosomes in a process that is dependent on PI 3-kinase [28] and that this process can be inhibited using LY294002, a specific and reversible inhibitor of PI 3-kinase [46]. We hypothesized that if the PMT-containing early endosomes, which also contain Tfn, fused with Rab5/EEA1-positive endosomes, then this fusion would be dependent on PI 3-kinase and therefore PMT trafficking could be prevented by treatment with LY294002. As shown in Figure 1E, pretreatment of HEK 293-T cells with LY294002 had no effect on rPMT-induced SRE-luciferase reporter activity at concentrations up to 75 µM and only a slight inhibitory effect at 100 µM; however, this concentration is twice that used to prevent the fusion of Arf6-positive and Rab5/EEA1-positive endosomes [28]. In control experiments, cell extracts were also blotted for phosphorylated Akt (P-Akt) and total Akt to demonstrate that LY294002 inhibited, as expected, phosphorylation and activation of Akt at concentrations of 10 µM or higher (Figure 1F). These results suggest that despite initial colocalization of PMTb with Tfn, a PI 3-kinase-mediated fusion event of Arf6-positive and Rab5/EEA1-positive endosomes is not necessary for PMT trafficking to a translocation-productive compartment.

### 3.3. Intra-Endosomal Acidification Is a Crucial Step in PMT Intoxication

Weak bases such as NH_4_Cl can be used to alkalinize endosomal pH [47]. Treating HEK-293T cells with 2 mM NH_4_Cl caused a dose-dependent decrease in PMT-induced SRE-luciferase reporter activity, with complete inhibition observed at concentrations above 5 mM (Figure 2A). Bafilomycin A1 (BAF), another specific inhibitor of endosomal acidification, slows the progression from early endosomes to late endosomes [27,48]. It was previously demonstrated that BAF inhibits PMT-mediated phosphorylation of FAK in Swiss 3T3 cells [20]. Similarly, when HEK 293-T cells were treated with BAF 15 min prior to treatment with rPMT, there was a dose-dependent decrease in PMT-induced SRE-luciferase reporter activity (Figure 2B). Taken together, the inhibition of PMT-induced SRE-luciferase reporter activity after treatment with BAF and NH_4_Cl demonstrates that acidification of the endosome is a crucial step in PMT intoxication of cells.

### 3.4. Colocalization of PMTb with CT B Subunit

In contrast to PMT, intra-endosomal acidification does not play a role in entry and translocation of CT [34,49], yet CT is present in Rab5-positive and Arf6-positive early endosomes and interacts with Arf6 during cellular intoxication [34,50]. A CT subunit B Alexa Fluor^®^-594 conjugate (CtxB-594) was added to the cells concurrently with PMTb-GFP and the distribution patterns were observed by confocal fluorescence microscopy after 3 h incubation in the absence or presence of 30mM NH_4_Cl (Figure 2C–J). In the absence of NH_4_Cl, PMTb-GFP showed a punctate localization that is spread throughout the cytosol and is colocalized with CTxB-594 (Figure 2C–F). In the presence of NH_4_Cl, the PMTb-GFP-containing endosomes localized to the periphery of the cell and did not colocalize with CTxB-594-containing endosomes (Figure 2G–J). Moreover, the distribution of CtxB-594-positive endosomes was not affected by treatment with NH_4_Cl. These results demonstrate that in contrast to CtxB, a toxin where intra-endosomal acidification is not thought to be important for entry, blocking intra-endosomal acidification with NH_4_Cl drastically affects the trafficking and localization of PMTb-GFP.

**Figure 3:**
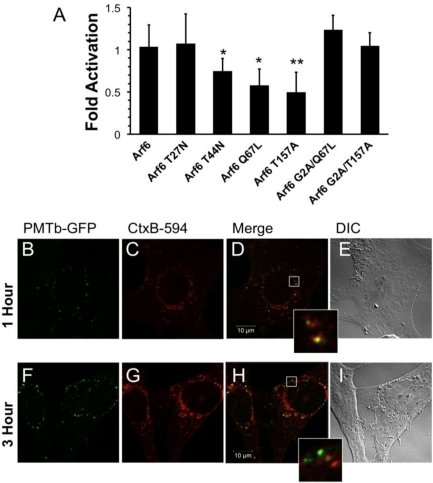
Arf6-dependent internalization and trafficking of PMT. HEK 293-T cells were transiently transfected with dual SRE-luciferase reporter plasmids, pcDNA3-Gα_q_, and plasmids containing Arf6 or Arf6 mutants, as described in Methods. Seven hours post-transfection cells were treated with 100 ng/mL PMT and incubated for 15 h before assaying for SRE reporter gene activity. SRE fold activation was determined by dividing SRE reporter gene activity in PMT treated cells by SRE reporter gene activity in untreated control cells. Fold activation was then normalized to empty vector control (pcDNA3). Data is expressed as an average of eight experiments ± S.D., with each experiment performed in triplicate, where * *p* < 0.05 and ** *p* < 0.005. (**b**–**i**) Swiss 3T3 cells were co-treated with PMTb-GFP and cholera toxin subunit B Alexa Fluor^®^-594 conjugate (CtxB-594) for 1 h (**b**–**e**) or 3 h (**f**–**i**). Cells were visualized by confocal microscopy. Panels (**b**) and (**f**), green channel image showing PMTb-GFP. Panels (**c**) and (**i**), red channel image showing CtxB-594. Panels (**d**) and (**h**), merged images of green and red channels. Panels (**e**) and (**i**), corresponding DIC images. Insets: Enlargement of the indicated section of the image, showing co-localization of PMTb-GFP and CtxB-594.

### 3.5. The Potential Role of Arf6 in Internalization and Trafficking of PMT

Previous studies show that intra-endosomal acidification by the V-ATPase results in recruitment of the small GTPase Arf6 and its GDP/GTP exchange factor (GEF) ADP-ribosylation factor nucleotide site opener (ARNO) to the endosomal membrane [22] where Arf6 binds to the c-subunit of the V-ATPase while ARNO binds to the a2-isoform [23]. This pH-driven recruitment of ARNO has been shown to be important for both receptor-mediated endocytosis and for trafficking between early and late endosomes. Since our results demonstrate that inhibitors of endosomal acidification, such as NH_4_Cl and BAF, inhibit PMT-induced SRE-luciferase reporter activity (Figure 2) and that PMT initially colocalizes with Tfn and CT in Arf6-positive vesicles, we hypothesized that the small GTPase Arf6 may play a role in internalization and trafficking of PMT.

To investigate the role of Arf6 in PMT trafficking, we overexpressed wildtype and several mutants of Arf6 to observe their effects on rPMT induced SRE-luciferase reporter activity. Arf6 is a small GTPase that relies on hydrolysis of a bound GTP and subsequent release of GDP to function properly. Therefore, any Arf6 mutants that have defects in GTP hydrolysis and subsequent GDP release will have impaired activity. The Arf6 Q67L mutant is a constitutively active mutant, while the Arf6 T27N and Arf6 T44N mutants are dominant-negative mutants. The Arf6 T157A mutant is constitutively cycling due to decreased binding of both GDP and GTP.

Overexpression of wildtype Arf6 had no effect on PMT-induced SRE-luciferase reporter activity in HEK 293-T cells treated with rPMT (Figure 3A), in agreement with previous studies that showed overexpressing wildtype Arf6 does not perturb the function, localization or distribution of Arf6 [24]. On the other hand, overexpression of constitutively active Arf6 Q67L or dominant-negative Arf6 T44N reduced the activity by 50% and 30%, respectively. Overexpression of the Arf6 T157A constitutively cycling mutant also resulted in 50% inhibition. These data strongly suggest a regulatory role for Arf6 in the internalization of PMT, presumably by preventing PMT receptor-containing vesicles from cycling to the surface or by preventing formation of PMT-containing endosomes. There was no significant effect of overexpression of the dominant-negative Arf6 T27N mutant on rPMT induced SRE-luciferase reporter activity. However, this mutant is less stable than the Arf6 T44N mutant [51], so the observed lack of effect of Arf6 T27N could be a result of instability. 

Next we wanted to determine if the effects of overexpression of Arf6 mutants on rPMT activity were dependent on the trafficking activity of Arf6. It was previously demonstrated that mutating the glycine at position 2 of Arf6 to alanine results in a cytosolic, nonmyristolated Arf6 that does not associate with membranes and is unable to mediate endocytic trafficking [52]. We hypothesized that if a mutant Arf6 that previously demonstrated inhibition of PMT-mediated SRE-luciferase reporter activity were mutated so that it could no longer associate with the membrane, then the ability of the mutant to decrease PMT intracellular activity would be abolished. To test this hypothesis, we chose the two mutants that showed the greatest inhibitory effect on PMT cellular activity, Arf6 Q67L and Arf6 T157A, and made an additional mutation substituting alanine for glycine at position 2 in order to abolish their myristolation, therefore preventing membrane association. Unlike their myristolated counterparts, the nonmyristolated Arf6 mutants, Arf6 G2A/Q67L and Arf6 G2A/T157A, did not block PMT-mediated SRE-luciferase reporter activity (Figure 3A). Taken together these results suggest that the inhibition of PMT-induced SRE-luciferase reporter activity observed with the Arf6 Q67L and the Arf6 T157A mutants is a direct consequence of their disruption of PMT trafficking through Arf6-containing vesicles inside the cell. 

To explore whether PMT may exploit a similar Arf6-dependent pathway for initial entry as CT [53], despite their differences in dependence on intra-endosomal acidification, we co-treated Swiss 3T3 cells with PMTb-GFP and CtxB-594 and observed the cellular distribution of both after 1 or 3 h by confocal fluorescence microscopy (Figure 3). After 1 h, PMTb-GFP and CtxB-594 were both observed in punctate vesicles localized throughout the cell (Figure 3A,B, respectively) and were largely colocalized (Figure 3D). After 3 h, the PMTb-GFP was still located in punctate vesicles throughout the cell (Figure 3F); however, co-treatment for 3 h resulted in divergence of PMTb-GFP from CtxB-594 (Figure 3H), with an accumulation of CtxB-594 to the perinuclear region (Figure 3G), while the PMTb-GFP remained predominantly localized in punctate vesicles throughout the cell (Figure 3F). These results are consistent with previous research demonstrating that CtxB-594 is trafficked to the ER [39]. These data suggest that while PMTb-GFP and CTxB-594 may initially share a common entry pathway, CtxB-594 is then trafficked to the Golgi and subsequently to the ER, while PMT remains in endosomal compartments, which are subsequently subject to intra-endosomal acidification and rendered capable PMT translocation.

### 3.6. PMT Trafficking Is Dependent on Actin Dynamics

**Figure 4:**
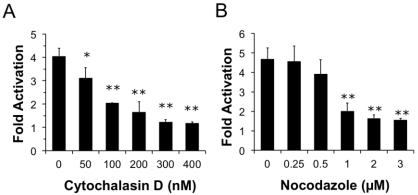
Both actin and microtubule dynamics are important for PMT trafficking to translocation-productive acidic late endosomes. HEK 293-T cells were transiently transfected with dual SRE-luciferase reporter plasmids and pcDNA3-Gα_q_ as described in Methods. Seven h post-transfection cells were treated with cytochalasin D (**a**) or nocodazole (**b**) at the indicated concentrations for 15 min before treatment with 100 ng/mL PMT. After 15 h incubation, cells were assayed for SRE reporter gene activity, as described in Methods. SRE fold activation was determined by dividing SRE reporter gene activity in PMT-treated cells by SRE reporter gene activity in untreated control cells. Data is expressed as an average of three experiments ± S.D., with each experiment performed in triplicate, where * *p* < 0.05 and ** *p* < 0.005.

We next wanted to determine whether components of the cytoskeleton are important for PMT entry and trafficking. We hypothesized that if actin dynamics play a prominent role in trafficking and internalization of PMT, pre-treating cells with cytochalasin D, an inhibitor of actin polymerization, would interfere with PMT trafficking pathways thereby inhibiting rPMT-induced SRE-luciferase reporter activity. To determine whether actin dynamics play a significant role in trafficking and internalization of PMT, HEK 293-T cells were pre-treated with varying concentrations of cytochalasin D for 15 min before rPMT treatment. As shown in Figure 4A, cytochalasin D treatment caused a dose-dependent decrease in PMT-induced SRE-luciferase reporter activity, with complete inhibition at concentrations over 0.3 µM, pointing towards the importance of actin dynamics for internalization and trafficking of PMT.

### 3.7. PMT Trafficking Is Dependent on Microtubule Dynamics

Microtubules are required for trafficking from early sorting endosomes to late acidic endosomes [27]. Consequently, we next investigated whether the PMT trafficking pathway is also dependent on microtubule dynamics. We hypothesized that if microtubule dynamics were important in the entry and trafficking of PMT, using an inhibitor of microtubule polymerization such as nocodazole would inhibit PMT-induced SRE-luciferase reporter activity. To determine if disruption of the microtubule network also disrupts PMT trafficking, we pre-treated HEK 293-T cells with nocodazole, a microtubule inhibitor, for 15 min. As shown in Figure 4B, pre-treatment of HEK-293T cells with nocodazole caused a dose-dependent decrease in PMT-induced SRE-luciferase reporter activity, with complete inhibition observed at concentrations of 1 µM or higher. Taken together these results demonstrate that both actin and microtubule dynamics play an important role in the entry and trafficking pathways of PMT.

### 3.8. Treatment with Brefeldin A Causes an Increase in PMT Activity

**Figure 5:**
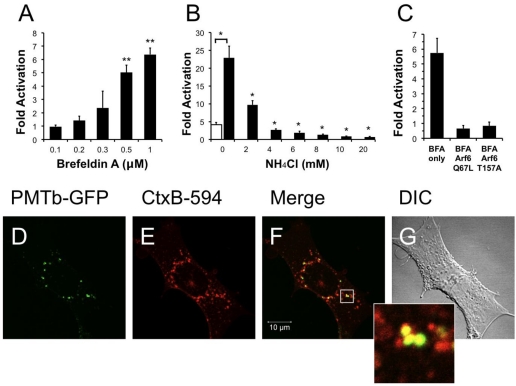
Treatment with brefeldin A enhances PMT activity. HEK 293-T cells were transiently transfected with dual SRE-luciferase reporter plasmids and pcDNA3-Gα_q_ as described in Methods. Seven h post-transfection cells were treated with brefeldin A (BFA) at the indicated concentrations (**a**) or with a combination of BFA (1 µM) and NH_4_Cl at the indicated concentrations (**b**) for 15 min before treatment with 100 ng/mL PMT. After 15 h incubation, the cells were assayed for SRE reporter gene activity as described in Methods. (**c**) HEK 293-T cells were transiently transfected with dual SRE-luciferase reporter plasmids, pcDNA3-Gα_q_, and plasmids containing Arf6 Q67L or Arf6 T157A, as described in Methods. Seven h post-transfection cells were treated with BFA (1 µM) for 15 min before treatment with 100 ng/mL PMT. After 15 h incubation, cells were assayed for SRE reporter gene activity as described in Methods. SRE fold activation was determined by dividing SRE reporter gene activity in PMT treated cells by SRE reporter gene activity in untreated control cells. Data is expressed as an average of two experiments ± S.D., with each performed in triplicate, where * *p* < 0.005. (**d**–**g**) The effect of BFA (1 µM) on the localization of PMTb-GFP and CtxB-594 in Swiss 3T3 cells after 3 h incubation. Cells were visualized by confocal microscopy. Panel (**d**), green channel image showing PMTb-GFP. Panel (**e**), red channel image showing CtxB-594. Panel (**f**), merged images of green and red channels. Panel (**g**), corresponding DIC image. Inset: Enlargement of the indicated section of the image, showing co-localization of PMTb-GFP (green) and CtxB-594 (red).

Brefeldin A (BFA) is a fungal metabolite that interrupts trafficking between the Golgi apparatus and the ER, leading to the accumulation of secretory proteins in the ER [40,54]. Treatment with BFA results in uncoating of the Golgi apparatus [55] and subsequent formation of tubulovesicular processes that redistribute the contents of the Golgi into the ER [56]. Although it was demonstrated that BFA treatment does not affect the distribution of Arf6 [24], BFA treatment does induce morphological changes in the distribution of early endosomes resulting in the formation of a tubular network similar to that seen during redistribution of the Golgi after treatment with BFA [57]. We next determined whether these morphological changes produced by BFA affected the entry and trafficking of PMT. Interestingly, pre-treatment of HEK-293T cells with BFA resulted in a dose-dependent increase in PMT-induced SRE-luciferase reporter activity (Figure 5A), with as much as a six-fold increase in PMT activity observed upon treatment with 1 µM BFA. These results suggest that the formation of a tubular endosomal system within the cell by BFA results in an increase in PMT activity.

We further explored this phenomenon by determining whether translocation of PMT from the endosome was necessary for the observed BFA-mediated increase in PMT activity. We hypothesized that if translocation from the endosome were necessary for the BFA-mediated increase in PMT activity, blocking translocation of PMT into the cytosol by treating with NH_4_Cl to prevent acidification of the endosome would effectively inhibit PMT activity. To test this possibility, HEK 293-T cells were pre-treated for 15 min with both 1 µM BFA and varying concentrations of NH_4_Cl up to 20 mM. As shown in Figure 5B, treatment of cells with as little as 2 mM NH_4_Cl significantly decreased the BFA-mediated increase in PMT activity, and indeed, at higher concentrations the inhibition by NH_4_Cl overrode the 5-fold activation observed in the absence of BFA. These data suggest that the BFA-mediated increase in PMT activity is dependent on translocation of PMT to the cytosol from acidic endosomes.

We then attempted to elucidate the mechanism of this BFA-mediated increase in PMT activity by determining whether this effect was occurring downstream of the Arf6 involvement in PMT trafficking. We first overexpressed in HEK-293T cells the Arf6 mutants, Arf6 Q67L or Arf6 T157A, which we found to decrease PMT activity by preventing PMT entry and trafficking (Figure 3A). The cells were then treated with 1 µM BFA for 15 min, followed by PMT treatment as before. As shown in Figure 5C, there was no increase in PMT-induced SRE-luciferase reporter activity in cells expressing either Arf6 Q67L or Arf6 T157A, as compared to cells transfected with an empty vector. These data suggest that the entry and trafficking of PMT into the cell via an Arf6 pathway occurs upstream of the BFA-mediated increase in PMT activity.

Next, we determined the localization of PMTb-GFP and CtxB-594 in cells pretreated with BFA. Swiss 3T3 cells were pretreated with 1 µM BFA for 15 min and subsequently co-treated with PMTb-GFP and CtxB-594 for 3 h, after which the cellular distribution of the toxin proteins were visualized by confocal fluorescence microscopy (Figure 5D,E, respectively). After treatment for 3 h, the PMTb-GFP and CtxB-594 appeared colocalized in large vesicles, predominantly near the perinucleolar region (Figure 5F). These vesicles appeared to be larger than the PMTb-GFP containing vesicles observed in the absence of BFA treatment (compare with Figure 2C–E).

## 4. Conclusions

This study further defines the trafficking pathways PMT uses to gain access to its intracellular targets. Previous studies showed that PMT inserts into lipid membranes and that this membrane insertion is pH-dependent [20] and occurs at a pH that causes PMT unfolding [21], suggesting that endosome acidification is important for the unfolding of PMT and its insertion into the membrane to form a translocation pore. In our experiments, we demonstrate that inhibiting endosomal acidification through the use of NH_4_Cl or BAF blocks PMT intracellular activity, as measured by PMT-mediated SRE-luciferase reporter activity. These results are in accordance with previous studies demonstrating that BAF inhibited PMT-mediated phosphorylation of FAK [20]. Interestingly, these earlier studies used BAF concentrations of 10–100 µM, a concentration range that is more than 1000-fold higher than the concentration used in the current study. Taken together these data indicate that intra-endosomal acidification is an essential step in PMT entry and translocation to the cytosol. Preventing intra-endosomal acidification blocks this translocation step, and instead PMT remains trapped in the endosome with a concomitant block in downstream SRE-luciferase reporter activity. Blocking intra-endosomal acidification also affected the distribution of the PMTb-GFP-containing vesicles as these vesicles were observed to accumulate near the membrane. In contrast, CtxB-594 trafficking was not affected as vesicles containing the CtxB-594 were distributed evenly throughout the cell. This would suggest that preventing intra-endosomal acidification not only blocks translocation and traps PMT in vesicles that remain near the cell membrane, but also affects trafficking of PMTb-GFP-containing vesicles within the cell.

It has been demonstrated that intra-endosomal acidification recruits the small GTPase Arf6 to the endosomal membrane [22]. Overexpression of the constitutively active mutant of Arf6, Arf6 Q67L, results in a dramatic morphological change as many membrane invaginations labeled with Arf6 Q67L develop [24] and increases cell surface binding of transferrin (Tfn), while decreasing the rate of Tfn internalization [32,33]. This suggests that while the mutation results in increased binding of the ligand, the protein is unable to subsequently internalize it. It is thought that GTP hydrolysis is essential for Arf6 function and the GTPase-deficient Arf6 Q67L mutant results in a block in endocytosis through Arf6-mediated pathways [30,58]. Accordingly, our results showing that overexpression of Arf6 Q67L decreases PMT-mediated SRE-luciferase reporter activity supports the notion that the mutation blocks PMT endocytosis through Arf6-mediated pathways.

Overexpression of the dominant-negative mutant of Arf6, Arf6 T27N, results in accumulation of the mutant Arf6 in tubulovesicle structures [24], suggesting that both GTP hydrolysis and release of GDP are necessary for Arf6 function. By disrupting the release of GDP, the dominant-negative mutant Arf6 T44N, which is more stable than the T27N mutant, should also disrupt the Arf6 trafficking pathway. When we overexpressed the Arf6 T44N mutant we observed a decrease in PMT-mediated SRE-luciferase reporter activity, further implicating the Arf6 pathway in the endocytic trafficking of PMT. The finding that the constitutively cycling mutant Arf6 T157A mutant, which binds, hydrolyzes and releases GTP faster than the wild-type Arf6 [59], also blocks PMT activity further supports these results. It has been suggested that Arf6 activation is responsible for returning recycling endosomes back to the plasma membrane [32]. Therefore it is possible that a quick cycling version of the mutant may quickly recycle endosomes back to the membrane thereby preventing acidification of the endosome. If the PMT-containing vesicles were recycled to the surface before the endosome is acidified, then PMT would be unable to translocate to the cytosol and hence show decreased activity. 

Membrane localization of Arf6 is mediated through its *N*-terminal myristolation, and a mutation at the myristoylation site (Arf6 G2A) results in subsequent localization of Arf6 to the cytosol [24]. As our results show, abolishing this myristolation in the Arf6 Q67L or Arf6 T157A mutants prevents the decrease in PMT-mediated SRE-luciferase reporter activity brought about by overexpression of the mutants. This demonstrates that the decrease in PMT activity is dependent upon association of Arf6 with the plasma membrane and upon Arf6-dependent trafficking functions, as cytosolic mutants of Arf6 showed no effect on PMT-induced SRE-luciferase reporter activity.

As previously mentioned, Arf6 is not only an allosteric activator of CT [35,36], but also has been implicated in trafficking of CT. Arf6 is recruited to the endosomal membrane in CT-intoxicated cells [34]. In addition, it has been previously demonstrated that Arf6 plays a role in uptake of transferrin [33]. If PMT were to exploit the same initial trafficking pathway as CT and Tfn, then one would expect to see PMT colocalized in vesicles with CT and Tfn. Our results show that after 1 h of treatment PMTb-GFP does colocalize with CtxB-594 and Tfn-TR in punctate structures throughout the cell, suggesting that PMTb-GFP uptake occurs through Arf6-containing endosomes. However, after treatment for 3 h PMT trafficking diverges from that of CT and Tfn, as evidenced by the failure of PI3-kinase inhibitor LY294002, which blocks fusion with Rab5-positive recycling vesicles that are used by Tfn, to block PMT activity and by the buildup of CtxB-594 in the ER, while the PMTb-GFP remains localized in punctate vesicles throughout the cell. These results support a model, whereby PMT, Tfn, and CT may share an initial trafficking pathway in Arf6 positive vesicles, but then these pathways diverge as CT is trafficked to the Golgi-ER and Tfn to recycling endosomes, while PMT traffics to an acidified endosome from which it can translocate.

Given the evidence that PMT, Tfn and CT diverge in their trafficking pathways, we wanted to further explore the trafficking pathways of PMT downstream of Arf6. Other toxins, such as DT, that exhibit a dependence on intra-endosomal acidification for translocation [60] have been shown to colocalize with Rab5 and early endosome markers such as EEA1 [61]. Studies have implicated the translocation domain of DT in the modulation of Rab5 activity [62]. Vesicles taken up by clathrin-independent endocytosis with Arf6 have been shown to recruit EEA1 to their surfaces before fusion with endosomes from other pathways, such as the Rab5 Q79L pathway. However, overexpression of Arf6 Q67L inhibits recruitment of EEA1 and subsequent fusion with other endosomal pathways [28]. This fusion of Arf6 and Rab5-positive vesicles was mediated by the activity of PI-3 kinase, as specific inhibitors of PI-3 kinase such as LY294002 prevent this fusion from occurring [28]. If PMT were acting in a manner similar to DT by exploiting Rab5 pathways for entry into the cell, it is conceivable that PMT/Arf6-positive vesicles would fuse with Rab5-positive vesicles in a PI-3 kinase dependent manner. However, the specific PI-3 kinase LY294002 had no effect on PMT SRE-luciferase reporter activity, despite decreasing phosphorylation of Akt, suggesting that the Arf6-Rab5 PI-3 kinase-mediated fusion of vesicles is not important for PMT trafficking and translocation. 

Not only have we shown that PMT is trafficked through a compartment with Arf6, we have also demonstrated the importance of the cytoskeleton in these trafficking pathways. Our results indicate that PMT trafficking is dependent upon both actin and microtubule dynamics, implicating both actin and microtubules in the trafficking pathways that PMT uses to enter the cell. To further investigate where the PMT and CT trafficking pathways diverge, we investigated the effects of brefeldin A (BFA) on PMT-mediated SRE-luciferase activity. BFA is a fungal metabolite that interrupts trafficking between the Golgi apparatus and the ER and causes accumulation of secretory proteins in the ER [40,54], uncoating of the Golgi apparatus [55], and subsequent formation of tubulovesicular processes that redistribute the contents of the Golgi into the ER [56]. Furthermore, trafficking through the Golgi cisternae is interrupted. It has been previously demonstrated that CT has a KDEL signal sequence [39] and that the toxin is trafficked in a retrograde manner from the Golgi to the ER, where it escapes to the cytoplasm to affect its intracellular target Gα_s_. Interrupting this retrograde trafficking pathway from the Golgi to the ER with addition of BFA blocks CT-mediated cAMP accumulation [41]. 

Our colocalization experiments demonstrated that, unlike CTxB-TR, PMTb-GFP was not trafficked to the ER. If PMTb-GFP were not trafficked to the ER, then BFA treatment would not be expected to affect PMT-mediated SRE-luciferase activity. However, we saw an increase in SRE-luciferase activity when cells were treated with BFA. Interestingly, VacA from *Helicobacter pylori* has also been shown to exhibit increased activity upon treatment with BFA [63]. There are several potential explanations for this increase. In addition to its effects on the Golgi apparatus, BFA induces similar effects on the endosomal system and causes the tubulation of both the endosomal system, as well as lysosomes. Although this mixed system is able to cycle between endosomes and the plasma membrane in a normal fashion, cycling between endosomes and lysosomes is impaired [57]. By impairing the pathway from the early endosome to the lysosome, PMT that may have otherwise been routed to a lysosome instead accumulates in the acidified endosome and translocates to the cytosol, resulting in the observed increased activity. Similarly, perhaps during the course of PMT trafficking, a fraction of the PMT is shuttled through the Golgi to the ER, where it is unable to escape to the cytosol. Blocking this non-productive trafficking through the use of BFA results in more PMT trapped in the acidic endosomes and thus available for translocation. 

However, other data suggests that while the morphology of the endosomal system is changed by BFA, cycling between the early endosome and the lysosome is not impaired [64]. If a block in trafficking to the lysosome is not responsible for the increase in PMT-mediated SRE–luciferase reporter activity brought about by BFA treatment, perhaps the morphological changes induced by BFA to form tubular endosomes results in the observed increase in PMT activity. It is possible that more PMT is able to translocate from the tubular endosomes, thereby resulting in an increase in PMT activity. Furthermore, studies have demonstrated that this BFA-induced tubulovesicle network associates with microtubules [65]. The association with microtubules is especially noteworthy as the decrease in PMT activity seen with nocodazole implicates microtubules as part of the trafficking pathway of PMT.

Alternatively, perhaps BFA treatment is responsible for increasing the number of PMT cell surface receptors. It has been shown that treatment with BFA increases the amount of cell-surface associated mannose-6-phosphate receptor (M6PR) [65]. Although a protein receptor for PMT is as of yet unidentified, it is possible that BFA induces an increase in the amount of a putative plasma membrane associated receptor, thereby allowing more PMT to bind to the cell surface and enter the cell via Arf6-mediated endocytosis. Further studies are needed to elucidate the mechanism of this BFA-mediated increase in PMT activity.

By overexpressing the Arf6 Q67L mutant that inhibited endocytosis, we were able to block the effects of BFA. This would suggest that the BFA mediated increase in PMT activity depends on PMT initially entering the cell. In addition, we showed that treating the cells with NH_4_Cl blocks the BFA mediated increase in PMT activity, indicating that translocation is necessary for this BFA-mediated increase in PMT activity. These results support the assertion that the increase in SRE-luciferase reporter activity after treatment with BFA is dependent on the ability of PMT to enter the cell via endocytosis and to translocate from the endosome. 

**Figure 6:**
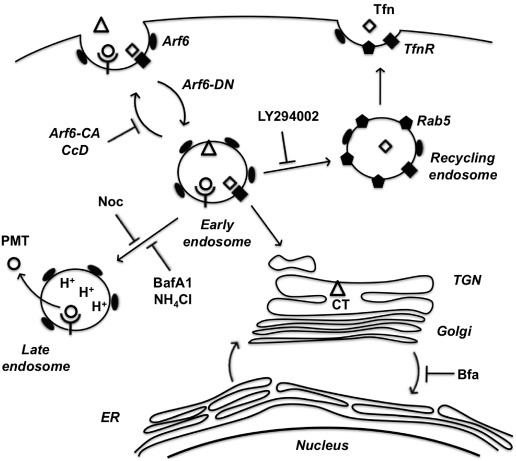
A model of PMT entry and trafficking. PMT enters the cell through an Arf6-dependent endocytic pathway that involves both actin and microtubule dynamics as evidenced by inhibition of PMT-mediated SRE activation upon expression of Arf6 mutants (Arf6-CA or Arf6-DN) or treatment with the inhibitors cytochalasinD (CcD) or nocodazole (Noc), respectively. PMT shares a similar route of endocytosis as transferrin (Tfn) and cholera toxin (CT); however, these pathways subsequently diverge. Tfn-bound receptors are trafficked to a Rab5-containing endosome in a PI 3-kinase-dependent vesicle fusion process, which is inhibited by LY294002. CT is trafficked retrograde through the trans-Golgi network (TGN) to the Golgi-ER. PMT is further trafficked through an endocytic vesicle that becomes acidified prior to translocation of PMT to the cytosol. Inhibitors of endosomal acidification, such as NH_4_Cl and bafilomycin A1 (BafA1), block the translocation of PMT, while brefeldin A (BFA) enhances this process by diverting any trafficking of PMT to the TGN instead to the acidified endocytic vesicles.

In summary, we have implicated the Arf6 trafficking route as a potential mode of entry for PMT. A model of PMT entry and trafficking that incorporates our findings as well as previous findings is shown in Figure 6. We have demonstrated dependence on intra-endosomal acidification for translocation and trafficking of PMT. These data suggest that PMT may share an initial entry pathway with Tfn and CT, but these pathways later diverge as Tfn is trafficked to Rab5-positive recycling endosomes and CT is trafficked in a retrograde fashion from the Golgi to the ER, while PMT translocates from an acidified late endosome. In addition, we have shown the importance of cytoskeletal dynamics of both actin and microtubules in PMT trafficking pathways. Finally, we have shown that BFA increases PMT activity, through an as-yet ill-defined mechanism that leads to more productive trafficking of the toxin to an acidic compartment for translocation.
